# Serial pathologic fractures of five long bones on four separate occasions in a patient with primary hyperparathyroidism, challenges of management in a developing country: a case report

**DOI:** 10.11604/pamj.2013.15.45.2501

**Published:** 2013-06-08

**Authors:** Samuel Adegboyega Olatoke, Olayide Sulaiman Agodirin, Ganiyu Adebisi Rahman, Olufemi Gbenga Habeeb, Rabiu Olusegun Jimoh, Bola Abdulkadir Ahmed, Sikiru Biliaminu, Olanrewaju Olubukola Oyedepo

**Affiliations:** 1 Division of General Surgery, Department of Surgery, University of Ilorin Teaching Hospital, Ilorin Kwara State, Nigeria; 2 Division of Orthopedics Surgery, Department of Surgery, University of Ilorin Teaching Hospital, Ilorin Kwara State, Nigeria; 3 Department of Chemical Pathology, University of Ilorin Teaching Hospital, Ilorin Kwara State, Nigeria; 4 Department of Anesthesia, University of Ilorin Teaching Hospital, Ilorin Kwara State, Nigeria

**Keywords:** Pathologic fractures, parathyroid adenoma, Primary Hyperparathyroidism, serum calcium, surgery

## Abstract

Multiple pathologic fractures secondary to parathyroid adenoma is rarely recognized and reported in the tropics. Inadequate evaluation causes worsened disability and increased psychological stress. We present a 27-year-old Nigerian male student with recurrent unexplained pathological fractures of the long bones. Primary Hyperparathyroidism was later diagnosed and he benefited from a unilateral parathyroidectomy. Primary hyperparathyroidism secondary to parathyroid adenoma is difficult to diagnose and needs a high index of suspicion. Surgery and good post-operative biochemical control of serum calcium produce satisfying outcomes.

## Introduction

Primary hyperparathyroidism (PHPT) is described as an abnormally increased parathyroid hormone (PTH) production from parathyroid gland(s) in relation to the serum calcium. It results from a disturbance of normal feedback control exerted by serum calcium [1]. Increased PTH production leads to hypercalcemia via bone resorption, increased gastrointestinal absorption of calcium, increased production of vitamin D3 and reduced renal calcium clearance.

PHPT occurs in 0.1-0.3% of the general population [1]. The exact etiology of the inappropriate increased secretion of parathyroid hormone is not known but the most commonly identified parathyroid pathology is the presence of a benign adenoma, other less common causes are, parathyroid hyperplasia and parathyroid carcinoma [1–4].

Occurrence of bone lesions such as brown tumors or osteitis fibrosa cystica and pathologic fractures in PHPT is now uncommon. It was reports to be about 10% in two large series [4–8]. Because of limited facilities in the developing countries, the diagnosis of PHPT is rare, rarer still is the diagnosis at asymptomatic state [9, 10]. Diagnosis usually follows overt presentation [6, 8]. We present one of such situations, a young man with multiple pathological fractures and osteitis fibrosa cystica.

## Patient and observation

A 27-year old Nigerian male student requiring surgical consultation for possible diagnosis of primary hyperparathyroidism. He had serial fracture of five long bones on four separate occasions, over a period of three and half years.

The first was fracture of the right humerus which was sustained from a fall while climbing a stairway in the dark. The second was fracture of the left femur which was sustained after a trivial unprovoked fall while walking a leveled ground (the first fracture had not healed on non-operative treatment before the second fracture despite an interval of about 6 months). The third was fracture of the left humerus, sustained while rehabilitating with a walking frame after surgery for the left femoral and right humeral fractures. The forth was a combination of right femoral and tibia fractures sustained simultaneously while bearing weight on the right lower limb as the leading limb to board a vehicle. Hyperparathyroidism was considered upon the forth occasion.

The history of weakness, generalized bone and joint pains preceded the first episode of fracture by about 1 year. He also had production of cloudy urine and history of epigastric pain diagnosed informally as gastritis from chronic ingestion of nonsteroidal anti-inflammatory He had previously had open reduction and internal fixation for the initial two fractures under general anesthesia He sustained mandibular fracture at intubation. He had been transfused 6 pints of blood on separate occasions. His genotype was AS, he was neither hypertensive nor diabetic. He was on chronic ingestion of calcium and vitamin D supplements. There was no history suggestive of associated thyroid disease and no family history suggestive of multiple endocrine neoplastic syndrome or primary hyperparathryrodism.

**Figure 1:**
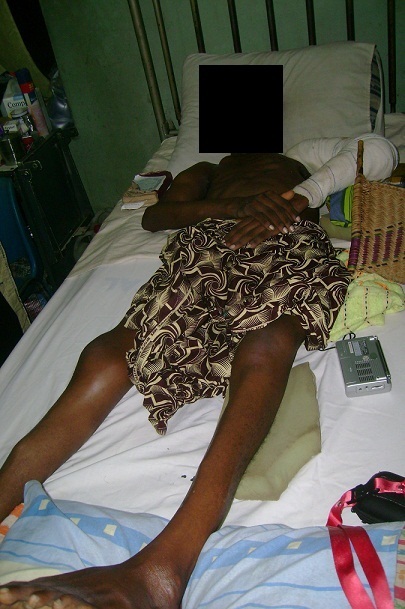
Physical findings at first contact with general surgeon

At first consultation by the general surgeon (Figure 1), physical examination revealed a young man, conscious and alert, in pains, wasted, with a global reduction in muscle bulk. There was frontal bursing and multiple bony swellings on the lower jaw, there was no clinical obvious neck mass. There were healed scars on the right arm and left thigh with reduced range of movement at both shoulders and both hip joints. On the right lower limb, the junction between the upper and middle third of the thigh was swollen, abnormally positioned and tender. There was midshaft swelling of the leg on the same limb, the swelling was soft and tender. There were no bruises or abrasions, and no external bleeding.

**Figure 2:**
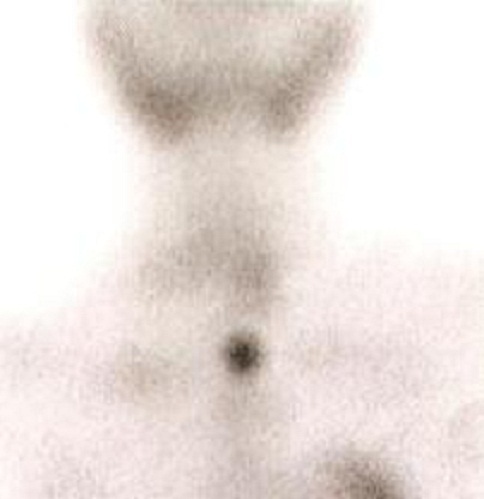
Sestamibi parathyroid scintigraphy revealed an increased focal uptake

**Figure 3:**
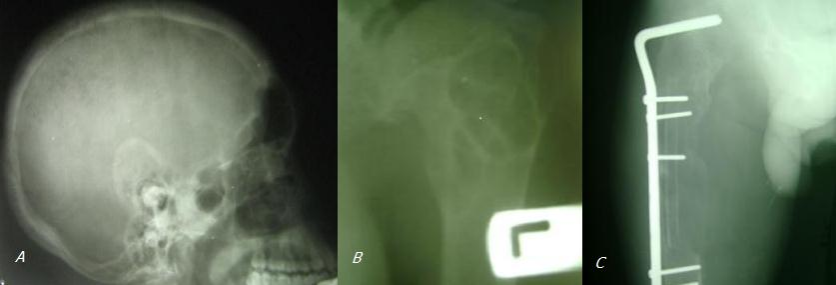
A) bony changes - salt and pepper appearance of the skull; B) bony changes - osteoporotic changes of the left humerous (with cystic changes on the head); C) bony changes - demineralized right femur (also shows fixed pathologic fracture)

Serum calcium and alkaline phosphatase were markedly elevated (3.17mmol/l and 260 I.U/l respectively). All other electrolytes and haemogram were within normal limits. Scouting X-rays revealed evidence of healed and healing fractures of the jaw and ribs in addition to the clinically obvious long bone fractures. Urinalysis showed 2+ haematuria. Neck ultrasound revealed an enlarged left lower parathyroid gland and the abdominal ultrasound scan showed multiple kidney stones. The serum parathyroid hormone level was 202.8 pmol/l (normal is 1.6-6.9 pmol/l). 99mTc Sestamibi parathyroid scintigraphy (Figure 2, Figure 3) revealed an increased focal uptake.

He subsequently had excision of the parathyroid mass (left inferior parathyroid) under cervical block and minimal sedation. The mass was grayish-black, measuring 7 by 4cm and weighed 32g. Histology revealed a parathyroid adenoma.

Postoperatively he had sudden and persistent symptomatic drop in serum calcium necessitating continuous infusion of calcium gluconate. He is currently 15 months post-surgery, he is being rehabilitated as an outpatient, he is pain free, more mobile and the serum calcium has remained normal (Figure 3).

## Discussion

In majority of the cases of PHPT, as was also seen in the presented patient, the Increased PTH production is from a solitary adenoma [2–4].

PHPT is one disease that is considered rare in developing countries because it is seldom diagnosed, but recent experience has shown otherwise [11]. Its apparent rarity in developing countries may be explained by the trio of limited diagnostic facilities, paucity of reports about the disease and outright non-performance of screening [3, 11]. When facilities are provided and screening is instituted more cases will be diagnosed and more reports will emanate from developing countries. Meanwhile a high index of suspicion is invaluable.

Our patient had been treated for pathologic fractures without determining the cause. The initial diagnostic workup did not include a search for hyperparathyroidism. The sex and age of presentation of this patient may have downplayed the diagnosis because primary hyperparathyroidism is more common in elderly females [1]. In addition the long duration of immobilization and sedentary habit and long duration of calcium supplementation were considered the explanations for the hypercalcemia detected at the initial diagnostic work up. The same were also considered as the cause of osteopenia demonstrated on X-ray. All these possible explanations of the findings notwithstanding, failure to consider hyperparathyroidism early in this patient's management was a massive omission and a lasting lesson in our clinical setting. However we must quickly add that in the developing countries it is not uncommon to overlook the diagnosis in patients presenting with some of the nonspecific symptoms until pathologic fractures occur while tin the developed countries florid bone lesions are mistaken for wide spread bony metastasis from a yet to be determined primary site [7, 8].

Our patient presented with florid features of bone complications, nephrolithiasis and overt biochemical derangement, These features are seen in developing countries and elsewhere where routine screening is not done in contrast to developed countries where most patients are diagnosed at early asymptomatic state because of screening facilities [3, 4]. In the “classic” clinical presentation which is no more commonly encountered, the patient with hyperparathyroidism presents with a pentad of, kidney stones, painful bones, abdominal groans, psychic moans, and fatigue overtones.

Clinically hypercalcemia with intact serum parathyroid hormone levels within or above the upper limits of normal and increased parathyroid cell mass suggest a diagnosis of primary hyperthryroidism [12]. The hypercalcemic component of clinical diagnosis though a prerequisite, may be absent in some patients. PTH assays can reliably distinguish PHPT from other causes of hypercalcemia. Parathyroid localization studies such as neck ultrasound scan and 99mTc Sestamibi scintigraphy are used to confirm the location of the diseased gland rather than for establishing the diagnosis of PHPT preoperatively.

Parathyroidectomy is the treatment of choice in PHPT. Success of the surgery is determined largely by early diagnosis and localization of the adenoma before surgery [13]. Intraoperative localizing and assessing investigations include quick PTH assay, gamma probes and frozen section [14, 15] none of this were available at our center. Operative approaches are either a unilateral or a bilateral neck exploration. Unilateral neck explorations have several advantages over bilateral, including reduced operative times and complications, such as injury to the recurrent laryngeal nerve and hypoparathyroidism, but there is a risk of missing another adenoma on the opposite side [16]. In our patient, having localized the lesion with ultrasound and sestamibi scanning, a unilateral exploration was done. Because there were no other indications for bilateral neck exploration and the patient was a poor candidate for general anesthesia with endotracheal intubation.

Post-operative after parathyroidectomy, the serum calcium resumes to normal levels within 1 to 3 days [17]. This is a welcome development and often gives assurance of success of surgery [17, 18] especially in situations where intraoperative or repeat PTH assay may not be achievable.

Hypocalcemia remains an important early complication of parathyroidectomy. This was demonstrated in our patient who developed features of recalcitrant hypocalcemia; carpopedal spasms on the forth postoperative day. He was treated with intravenous calcium gluconate infusion for over 8 days. The drop in serum PTH was reassuring to us but the persistence also presented a management challenge. This sustained and prolonged postoperative hypocalcemia has been documented [17].

The pathogenesis of the sudden and persistent drop in the parathyroid hormone: The diseased hyperfunctioning parathyroid gland suppresses the other glands such that its excision means removal of the sole source of the parathyroid hormone at the time. Consequently, the PTH-induced osteoclastic bone resorption stops while the osteoblastic activity continues resulting in a marked increase in bone uptake of calcium and phosphate (the hungry bone syndrome). This complication should be anticipated preoperatively and preemptive management instituted [18]. The preventive approach or pre-emptive treatment will be less distressing for the patient and less tasking for the physician. The presence of oseogenic symptoms, elevated serum alkaline phosphatase, long duration of the disease and the size of the gland may be predictive values for postoperative hypocalcemia [18].

Post resection confirmatory test of the sestamibi and PTH assat were not done in this instance because of the cost and the logistics. The preoperative sestamibi test was done about 250km away while the PTH assay was done in South Africa. The non-availability of this diagnostic facilities in our center contributed to the delay in the diagnosis and treatment and discouraged a post-operative repeat. Our patient was last reviewed in the surgery outpatient department 6 months ago. There were no new osteogenic symptoms and there has been sustained improvement in his musculoskeletal function

## Conclusion

Diagnosis of PHPT requires a high index of suspicion, hence patients presenting with pathological fractures should have routine serum calcium. A combination of biochemical tests, including serum levels of calcium, phosphorus, alkaline phosphatase and PTH assay will help in diagnosing PHPT in large number of cases of the cases. Parathyroidectomy done after adequate localization, aggressive calcium supplementation/replacement and either open or closed management of fractures ensures adequate fracture healing.
